# The Creative Awareness Theory: A Grounded Theory Study of Inherent Self-Regulation in Attention Deficit Hyperactivity Disorder

**DOI:** 10.3390/jcm13195963

**Published:** 2024-10-07

**Authors:** Rebecca E. Champ, Marios Adamou, Warren Gillibrand, Sally Arrey, Barry Tolchard

**Affiliations:** 1 School of Human and Health Sciences, University of Huddersfield, West Yorkshire, Huddersfield HD1 3DH, UK; marios.adamou@swyt.nhs.uk; 2 Department of Nursing, School of Human and Health Sciences, University of Huddersfield, West Yorkshire, Huddersfield HD1 3DH, UK; w.p.gillibrand@hud.ac.uk (W.G.); s.ketchenarrey@hud.ac.uk (S.A.); 3 Nursing and Midwifery, School of Health and Life Sciences, University of Teesside, North Yorkshire, Middlesbrough TS1 3BX, UK; b.tolchard@tees.ac.uk

**Keywords:** adult ADHD, neurodevelopmental disorders, self-regulation, grounded theory, self-determination theory, positive aspects, positive attributes, creative adjustment

## Abstract

**Objectives:** The aim of this study was to determine why and how adults with attention deficit hyperactivity disorder (ADHD) experience variable impairment and identify the processes and strategies adults with ADHD use to develop positive self-regulation skills. **Methods:** A mixed cohort of 11 participants (6 female and 5 male) from a university, an adult ADHD clinic and an ADHD support group in the UK were interviewed online between September 2021 and February 2022. Data were collected and analysed simultaneously, inspired by a constructivist grounded theory methodology. **Results:** Participants described a “polar awareness of difference” from others in terms of engagement and ADHD characteristics, and a “polar awareness of consciousness” experienced as the states of *chaotic attention* and *hyperfocus*, both of which impact core perceptions of self. Using an infinity paradigm, the results demonstrate unskilled attempts to self-regulate within and between these states using *self-absorption* or *self-transcendence* strategies, including their inherent challenges and energetic cost. Our results further indicate that at the centre of this dynamic paradigm, *creative awareness* strategies exist, which exemplify polarity awareness and the regulation of that awareness supported by an authentic inner compass (AIC). **Conclusions:** This paper presents the empirical foundation for the ADHD Creative Awareness Theory (CAT)—a new theory for understanding the experience of ADHD consciousness and environmental engagement. Practical implications are explored, and recommendations include use of the CAT as a framework for understanding and development of inherent self-regulation skills for adults with ADHD.

## 1. Introduction

The impact of cognitive behavioural theory on both research and treatment design for ADHD has resulted in a singular deficit perspective of the theoretical origins of ADHD [1]. This perspective is limiting in terms of positive outcomes for treatment and perpetuates a focus on symptom reduction and maladaptive behaviours. While ADHD is primarily viewed as a neurobiological disorder, the possibility that ADHD may confer advantages to the individual is widely debated [2,3,4,5,6,7,8,9,10,11]. The characterisation proposed by cognitive behavioural-based theoretical models highlights that the executive function (EF) deficits and impairments of ADHD are such that it should be considered a chronic mental disorder with no cure, and treatments should specifically focus on pharmacological support, reducing stigma and building self-protective attitudes of resilience, self-esteem, and self-efficacy [4,12,13,14,15,16,17]. This is supported by psychoeducation to develop a perception of internal “resources” that can assist with daily coping and dealing with impairments, goal achievement and developing perceived self-efficacy [18,19,20,21]. Resources are defined as individual characteristics which strongly engage a patient’s motivation and are important for self-esteem, such as goals, values and possibilities. These can be mobilised for a process of change in therapy known as “resource activation” [22]. In ADHD, these are identified as personal competence, strengths and aptitudes that may have been stifled or sacrificed due to impairments, which are important in developing strategies for symptom control [23,24,25].

Academic research using the cognitive behavioural theoretical characterisation of ADHD has supported only the concept of “individual strengths” and has stated that no study has conveyed any universal strengths or advantages associated with the neurobiology of ADHD beyond that of control groups [12,17,26,27,28,29], despite a large growing body of anecdotal work regarding positive aspects of ADHD [2,5,30,31,32,33,34,35,36,37]. While resource activation is undoubtedly useful to the development of the self-esteem of the individual, recent research suggests there may be more universal positive attributes that accompany the neurobiology of ADHD including hyperfocus, curiosity, energy, courage, flexibility, humanity, resilience, entrepreneurship, transcendence, divergent thinking, and creativity [6,8,11,38,39,40,41,42,43,44,45,46,47,48]. Research on strengths and resources of ADHD has previously been based primarily on qualitative and phenomenological methodologies [8,24,30,47,49] and thematic analysis [50], with the exception of quantitative research on measures of creativity and divergent thinking [11,38,39,40,41,51]. Three grounded theory studies were identified; however, all three studies were based on the cognitive behavioural characterisation of ADHD, and the focus was the experience of late diagnosis [25], the advantages of late diagnosis [52] and quality of life with ADHD [53]. There is a paucity of research in this area in general, and in particular from a perspective not based in cognitive behavioural theory.

Self-determination theory (SDT) has been suggested as an alternative theoretical perspective through which to understand the origins, motivations and behaviours associated with ADHD [54]. Viewing ADHD behaviours through a different theoretical lens provides an opportunity to explore not only the origins of those behaviours but also other assumptions associated with its characterisation [1]. The focus of this study was to identify universal selective strengths that support individuals with ADHD to manage the variability of their impairment. The aim of the study was to generate a theory, grounded in data, that explains (a) why and how individuals with ADHD experience variable impairment; (b) the impact of variable impairment for those with ADHD; and (c) processes and strategies used by those with ADHD to resolve their main concerns regarding the impact and consequences of the variable impairment of ADHD.

## 2. Materials & Methods

### 2.1. Study Design and Data Collection Tools

#### 2.1.1. Method

##### Philosophical Framework

This study has adopted a pragmatic approach in line with the philosophical framework of this thesis. Research on phenomena concerning the experience of ADHD has theoretical implications, and grounded theory is presented as a methodology that is ideal for an area where not much research or theorising has been carried out before [55]. This project employed a constructivist approach [56] which combines features of the Glaser and Strauss [57] methodology, including the constant comparative method, and the Strauss and Corbin [58] methodology, including abductive reasoning; this project was guided by Flick’s [55] systematic phases of grounded theory: initial, conceptual–theoretical, confirmatory–selective, and reflective. A constructivist approach recognises the subjectivity and researcher involvement and interpretation of data in theory construction [56]. This was thought to be an important element to monitor during the research process due to the researcher’s own personal experience of ADHD.

##### Methodological Approach

Grounded theory methodology is a dynamic process which includes successive iterations of theoretical sampling. This requires the researcher to adopt an intuitive stance at the beginning of the research, which was considered essential by Glaser to allow the theoretical structure of the issue being studied to emerge [59]. This study followed a systemisation of the intuition process inherent in grounded theory as presented by Flick [55]. He outlines several phases in the grounded theory research process, which are described herein.

#### 2.1.2. Initial Phase

Because data collection methods flow from the research question rather than driving it [56], flexibility in data collection methods throughout the research is encouraged, giving grounded theory a more ethnographic approach than some traditional qualitative methods. Sensitising concepts, or initial ideas, interests and questions to pursue can guide researchers to places at which to start inquiry [56]. This study used memos, which is a key recommended data collection method begun during this phase; memos document the experience of the research process including coding ideas, reflections, diagramming of concepts, and reviews in order to advance abstract levels of theorising [55,60,61]. Initial sampling of data was untangled, or segmented, into single words or short sequences of words to generate units of meaning defined as concepts or ‘codes’. The constant comparative method, or comparing data to data, was selected for this study to direct coding categorisation, and the process followed constructivist methodology, wherein this coding is emergent and conducted line by line.

#### 2.1.3. Conceptual–Theoretical Phase

This phase saw initial sampling become theoretical sampling, where cases, groups or materials are sampled according to their relevance to the developing theory. Two types of coding in constructivist methodology occur in this phase, focused and theoretical, where the most significant or frequent codes that make the most analytical sense are identified. These were assessed for relevance and potential to become conceptual categories, and relationships between categories were also identified. This phase also saw an analysis shift from inductive logic to abductive reasoning. Constructivist theory highlights the importance of this shift, as abductive reasoning aims to account for surprises, anomalies, or puzzles in the collected data, which may take the researcher into unanticipated directions or theoretical realms [56]. Awareness of this particular aspect of the constructivist approach proved key in facilitating a significant shift in the analysis process in this study. This progressed iteratively into theoretical coding or conceptualising how codes relate to each other and integrate to form a theory. Charmaz [56] uses Glaser’s [62] ambiguity regarding codes both as classifiable and emergent from the research as a tension between the data and inspirational sources of existing concepts and theories to add precision and clarity to the analysis. In this study, this provided a flexibility between establishing static codes and remaining responsive to new concepts arising from the data. This phase also included sorting and diagramming memos according to the developing theory, which were used to elaborate upon and refine categories to guide theoretical sampling and coding toward integration.

#### 2.1.4. Confirmatory–Selective Phase

As categories became increasingly abstract and demonstrated more theoretical connections, the coding process became more selective to confirm the centrality of categories and their relevance to the developing theory. The focus of this phase in this study involved dynamically shifting between categories, with further sampling used as a strategy for identifying variation and gaps among categories.

#### 2.1.5. Reflexive Phase

When new data provide neither new properties for a category nor further insights into the theory, it may be that theoretical saturation has been reached [55,56,62]. In this study, theoretical saturation was identified by clarity in links between the relationships and concepts developed in the research process. Theoretical sorting of memos provided a basis for creating, refining, and organising theoretical links for writing.

### 2.2. Data Collection and Analysis

Unlike other qualitative methods, grounded theory approaches recommend that the inquiry shapes the data collection [56,57]. This process of finding and generating data that is “rich”, or detailed, focused, and full, provides solid material for substantive analysis. For this project, data were provided from intensive interviews and memos or field notes by the researcher.

#### 2.2.1. Intensive Interviews

A key difference in constructivist grounded theory is its approach to interviews. Charmaz’s [56] style, called intensive interviewing, is defined as “a gently guided one-sided conversation that explores participants’ perspective on their personal experience with the research topic” (p. 56). Intensive interviewing uses a similar attitude to grounded theory in the approach of the researcher; it is open-ended but directed, shaped but emergent, and paced yet unrestricted [56]. The intensive interviewing approach can elicit a dialectic exchange of responses and discourses from the participant, highlighting not only concerns and reflections, but also identities and social connections. This provides the researcher with a chance to enter the participant’s implicit world of meaning, facilitating insight through emergent connection and sharing of human experience. Questions are shaped using open questioning and a Rogerian [63] non-directive client-centred approach including participants’ own language to encourage further expression and detail. Participant language and meaning is key in constructivist grounded theory, alerting the researcher to the impact of the co-creation of data and the influence of their role in the process. The key characteristics of intensive interviewing are as follows:

- Selection of participants who have first-hand experience;
- In-depth exploration of participants’ experience and situations;
- Objective of obtaining detailed responses;
- Emphasis on understanding participants’ perspective, meanings, and experience;
- Practice of following up on unanticipated areas of inquiry, hints, and implicit views and accounts of actions [56].

#### 2.2.2. Memos or Field Notes

Memo writing is an essential part of the grounded theory research practice. While the style and structure of memo writing is left up to the individual researcher, it is seen as an essential practice in conceptualising the data for analysis [55]. Memos provide a record of the researcher perspective and the research process and assist in the review and identification of gaps that need investigation [56]. Ideally, memo writing will also bring transparency to the research process, acting as a research diary that is begun from the initial stages. Therefore, this project will include memo writing as additional data for analysis.

#### 2.2.3. NVivo

Grounded theory has some significant methodological differences in its approaches due to varying epistemological views. This can cause confusion for researchers and lead to some quality control issues in research [64]. Therefore, the ability to present a transparent account of the research process is recommended [64,65]. The use of a computer-assisted qualitative data analysis software program (CAQDAS) can not only enhance the data handling and analysis process but also increase the effectiveness of the process of learning from the data if the software is effectively utilised for the project [65,66,67]. NVivo has been demonstrated to support and facilitate the iterative process that is core to grounded theory and to provide the process transparency that is recommended to enhance study validity [64]. Therefore, this project will use NVivo 20 (Lumivero, Oxford, UK) from the initial phase to coordinate, organise, and manage all data as well as utilise the program for conceptual and theoretical development.

#### 2.2.4. Ethics and Permissions

Ethical approval was sought from the University of Huddersfield School of Human and Health Sciences—School Research Ethics and Integrity Committee (SREIC) and a regional NHS Research Ethics Committee, since participants were recruited from an NHS trust, universities, and the community. All sessions with participants were held in adherence with the Ethical Framework for Good Practice set by the UK Council for Psychotherapy (UKCP) [68].

The ethical implications for this study were considered in the following contexts.

#### 2.2.5. Pre-Study Preparation

A critical element of this research is the exploration and representation of the lived experience of adults with ADHD, and the applicability, efficacy, and accessibility of the results. Therefore, a patient participation group was organised in pre-study preparation before recruitment. A small group of 5–6 adults with ADHD who expressed strong interest in the research reviewed and refined recruitment materials including the patient information sheet, consent form and interview guide.

#### 2.2.6. Data Protection and Data Storage

The researcher is expected to comply with and update their knowledge of the requirements of the General Data Protection Regulation (GDPR), the NHS Confidentiality Code of Practice, the Computer Misuse Act (covering information security), and all Local Trust Policies with regard to the collection, storage, processing and disclosure of personal information and also to uphold the core principles of data confidentiality both in letter and in spirit. All participant case records were held in accordance with the Data Protection Act [69]. To protect the identity of individual participants, all personally identifiable data (PID) were anonymised and will not be released. Information on confidentiality policy and anonymisation of PID was included in the consent form. Records were kept both in electronic (consent forms, agreements) and hard copy form (memos, process analysis). If electronic access was unavailable, participants were sent hard copies which were scanned electronically, and the originals were destroyed. The participants’ home addresses (including postcodes) and telephone numbers were kept on a secure database and spreadsheet on NHS/university computers in compliance with the Data Protection Act [69]. Data held in the NVivo database were anonymised and only accessible by password-protected researcher login. All manual records were kept in a locked cabinet accessible by the researcher only. In accordance with the Data Protection Act [69], personal data will not be retained for longer than is necessary. All participant personal data, transcripts, recordings, memos, and process notes will be retained in order to obtain permission for the study results to be published, in accordance with ethical approval.

#### 2.2.7. Sampling and Participants

Traditional sampling in quantitative research aims to identify a ‘primary selection’ of cases who possess the optimum knowledge or experience about an issue and the skills to communicate about it effectively. This then becomes a basis for further sampling and generalisation to a wider population [70,71]. As we have seen, the sampling process is quite different for grounded theory research, where simultaneous sampling and coding create an initial category set. In-keeping with the focus on selecting cases which may provide new insights for developing theory, it was considered important to gather data from cases with a comparatively wide range of experience including age, gender, geographical location, and how recently diagnosis was received. Participants were recruited from three different participation groups: NHS patients from the Adult ADHD Clinic at the South West Yorkshire Partnership NHS Foundation Trust; university students at the University of Huddersfield and the University of Cambridge; and an ADHD Support Group for adults with ADHD. Inclusion and exclusion criteria were as follows.

#### 2.2.8. Inclusion Criteria

- Confirmed diagnosis of ADHD;
- Age 18 or older;
- Access to computer or smartphone with an internet connection.

#### 2.2.9. Exclusion Criteria

- Comorbid diagnosis (e.g., autism, bi-polar, intellectual disabilities, learning difficulties, traumatic brain injury, psychosis or Tourette’s);
- Substance abuse disorders;
- Other mental health disorders (e.g., PTSD, oppositional defiant disorder);
- Personality disorders.

In total, 13 participants (6 female-presenting and 7 male-presenting between the ages of 20 and 52) participated in the interviews. Participants were screened by the researcher for exclusion criteria through initial email contact and live at the online interview stage. Two participants in the initial phase (2 and 5) were identified as having comorbid diagnoses at interview and had to be excluded from the study. Of the remaining 11 participants, one was Hispanic American, one South American, and the remaining 9 were White British. One participant received a diagnosis in childhood, and 10 received a diagnosis in adulthood, between the years of 2011 and 2021. Subgroups of diagnosis were also divided, with 7 participants identifying as inattentive type and 4 identifying as combined type (See Table 1).

Sites involved in the project made information available to their respective populations but did not engage with active recruitment (e.g., post fliers or information regarding the study in groups or newsletters). Patient information sheets and letters of consent were provided to participants who contacted the researcher directly with an interest in participating in the study. An interview guide was generated as part of the research approval process and as an example to show potential participants. Due to COVID-19 pandemic restrictions on face-to-face contact, all interactions with potential participants were conducted online through NHS-approved e-signature platforms, as agreed by the HRA and MHRA [72].

### 2.3. Procedure

Intensive interviews were conducted online between September 2021 and February 2022. Participants received an email invitation to an initial interview to discuss participation in the study. Participants were offered a 60–90 min online semi-structured interview with the researcher to explore their experience of living with ADHD. In addition, all interview participants were entered into a draw for a £50 Amazon gift voucher. Consent forms were completed live during the session via a shared document file to provide the opportunity to ask questions.

### 2.4. Data Analysis

Each interview was transcribed and uploaded into NVivo for line-by-line coding alongside the video to ensure clarity. This generated several initial themes, which were consistent across interviews. Analysis of the data was an iterative process using the constant comparative method, moving between the transcripts, writing memos, diagramming codes to develop themes, linking themes together, and arriving at core concepts. A theoretical model was identified at the cessation of the 7th interview, and a further 4 interviews were completed to confirm the model. The initial phase produced 70 codes from all 7 interviews, which were grouped into 5 themes. Review of these themes in the conceptual–theoretical phase led to the emergence of surprising core themes, which resulted in reviewing the transcripts to code to new emergent themes. This generated 38 codes, leading to a final 7 core themes and 35 subthemes in the confirmatory–selective phase, and the process was repeated until no further themes emerged and saturation was reached in the reflexive phase.

## 3. Results

The theory emerging from this process is termed the ADHD Creative Awareness Theory (CAT). The ADHD CAT uses a self-determination theory (SDT) [73]-based framework as the primary theoretical perspective for characterising ADHD motivation and behaviour [54]. Using the example of an infinity paradigm, the ADHD CAT describes the polar nature of ADHD self-awareness and identifies the strategies and processes ADHD individuals are engaging in to successfully self-regulate their experiences. The theory identifies two distinct areas of polar awareness, challenges involved in the management of these states, and the skills and resources that contribute to positive self-regulation. A data sample for each theme is presented here, and a more complete sample is available [74].

### 3.1. Polar Awareness of Difference

#### 3.1.1. Environmental Engagement

Previous research confirms that individuals with ADHD are aware of a difference between themselves and others. However, this has been reported as a sense of feeling “different” or socially unaccepted due to difference in or comparison of capability [8,23,24,75]. SDT highlights the principle of organismic integration, or an active process of seeking engagement with the environment, as a fundamental component of internalisation and development of the self-concept [73,76]. Participants described a long-standing and ongoing awareness of different experiences of the process of engagement with the environment from others. They describe their natural approaches to engagement as confusing to others, leading to stigma, misunderstanding and negative responses to behaviours. Additionally, participants described experiencing a greater amount of effort to successfully engage with their environment in ways similar to others or meet social standards as compared to others, which often resulted in frustration, exhaustion and feelings of resentment.

Participant 3: “I think that the first difference would probably be the way I will react to certain things or the way my body clock runs… But I think that there is a stigma of that whole situation, if you get up late and go to bed late, that inherently is lazy. Even if you’re up for the same amount of time as someone that will get up earlier and go to bed early and I think that potentially caused some clashes and understanding with my family. And also my reactions to things will be very different to them. Certain things that might seem quite trivial to my family are often more amplified in my emotional reactivity about the situation”.

Participant 6: “Everyone does this to some degree, but it feels like it’s harder for me… I try and remember that it is in some capacity acknowledged that it is more difficult for people with ADHD to do some things, especially in academics, in terms of keeping on track and in self-motivation, that kind of thing, with no structure. And I can acknowledge how difficult it must be to take yourself… that far. So, I think it takes a lot more effort on my part to start working ahead of a deadline than it does for anyone I know… it’s much more of an emotional upheaval for me to sit and start writing an essay that’s due in three days. My peers get stressed out when they haven’t started something… due in a week. It’s not even on my radar if it’s due in a week… it’s first draft is final draft. I will proofread it in the 10 min before it’s due. And that’s an advantage that they have”.

Participant 10: “One of the problems I’ve got with that is I either go one way or the other. If I’m waffling, I’m not going to get the interest I need from the people I need to kind of get on board with the idea. When I’m concise, what I’ve said makes perfect sense to me, but other people can’t make the connections I’ve made to make it make sense. So then, I’m kind of stuck because in my head I’ve just, all I’ve said is grass is green and they’ve gone, “What?” And I think, well, hang on a minute. What I’ve said was so simple. I couldn’t mean anything else by that. And then with a bit of a back and forth… I can start to understand, you know, the lack of connections they’ve made. Now… I’m making the most ridiculous connections, but they make sense to me because there is a very clear-cut link, but I can really feel in those moments that I’m just thinking in such a different way from everyone else”.

#### 3.1.2. Positive Characteristics of ADHD

Conversely, participants felt some of their natural ways of engaging with the environment were helpful and beneficial. The themes of *empathy, curiosity, divergent thinking*, and *humour* were all perceived as characteristics associated with participant’s own experience of ADHD or as a shared experience with other ADHD individuals, which is supported by previous research [8,11,38,39,40,41,45,51].

Participant 3: “I think that everyone I’ve met with ADHD has a lot of empathy abilities. I think that’s quite good for people’s skills and understanding and being able to put yourself in people’s shoes, no matter what that is. Obviously speaking on behalf of myself from like acting-wise or theatre wise, writing, to things like coaching or counselling and just people-based, like social care people-based jobs. I think we’re very good at those sorts of things“.

Participant 4: “I would say the positive side would be that it helps me see patterns that would-that no one ever thought about, like the people when I meet them or talk to them, they wouldn’t realize that to be like, oh, that’s interesting. I’ve never thought of it that way. Like that’s something cause with ADHD my mind jumping to so many different things, I’m able to see patterns and connections that I would say, someone else, maybe… maybe would miss those. So even with that I would say it might be an advantage.”

Participant 7: “I’m a very curious person by nature and I think that curiosity plays a big role in my motivation, which in curiosity, and like interests are almost, they live, in a large overlap, like things I’ll be curious about are obviously going to be the things I’m interested about. Kind of asking more questions to myself about the problem I’m trying to solve can help me gain interest in the problem because of, they will build, like, if I’m curious about the problem, which means I’m asking questions about it, questions about like details that might not be so obvious or given to me in the beginning”.

Interestingly, while they were identified as positive aspects of their experience, none of the participants attributed successful management of their ADHD to these positive characteristics individually. It was this surprising finding that triggered the re-examination and recategorization of the data in relation to ADHD experience of engagement with the environment at the conceptual–theoretical phase (Figures S1–S4).

### 3.2. Polar Awareness of ADHD Consciousness

The experience of two states of consciousness in ADHD emerged from the data: *chaotic attention* and *hyperfocus*. *Chaotic attention* is defined as “rapid chaotic movement between unrelated and unconnected thoughts and ideas”. Also known as mind wandering [77], participants described experiencing a “busy brain”, “monkey mind”, “scattered thoughts”, and “mental noise”. It is characterised by *variable concentration*, or continual engagement and disengagement with the environment or internal thoughts; *exploration*, or engagement with novelty, new learning, and making mental leaps or connections; and *emotional responsiveness*, or emotional expression appearing disproportionate to context. Participants described this state as impairing goal-directed behaviour due to feeling unable to control levels of focus and engagement, resulting in a lack of consistency in completing tasks, difficulty being present in conversations, and inability to maintain engagement if interest is not present. The effort required to engage with some activities while in this state sometimes results in strong emotional responses. In general terms, *chaotic attention* appears omnipresent; however, in relation to goal-directed behaviour it is often accompanied by negative feelings of frustration, overwhelm, uncertainty, lack of motivation, and emotional reactivity.

Participant 4: “It’s like, I, I know that theoretical understanding that, okay, my frontal cortex lacks enough dopamine to kind of properly have an advanced plan and to do things on time, but there may be other things. If that lack of dopamine might be the reason why I always seek out different novelty or to do different things, that can be an advantage if it’s applied in the correct way, because it keeps something exciting. It keeps that subject exciting rather than just boring and kind of get it done, kind of gives new life to that subject.”

Participant 13: “And you know it can be difficult just to control your mood and your feelings and emotions. Certainly, for me, it presents a lot of anxiety with that, which is probably more so from other people’s views, you know the typical comments of, ‘lazy, can’t be bothered, doesn’t apply themselves properly. If only she’d apply herself.’ You think, ‘if only know you knew’”.

Participants contextualised *chaotic attention* by placing it in contrast with the polar opposite state of *hyperfocus*. *Hyperfocus* is defined as “a driven, intense, narrow, concentrated focus”. Participants described this state as “tunnel vision”, “inertic”, “in the zone”, and “plugged in”. It is characterised by *engagement, immersion, connection, flow* and a sense of *awe*. Participants described this state as becoming deeply and passionately engaged, resulting in intense bursts of productivity and a positive sense of alignment of attention. *Hyperfocus* is accompanied by feelings of both excitement and calm, inspiration, thriving, dedication, joy, and accomplishment. *Hyperfocus* is also associated with interest and the ability to use and store information while engaged. However, this state was described as impairing due to its apparent randomness and sense of compulsion.

Participant 4: “When I hyperfocus, I feel like I’m in a different zone, like a different universe almost. My mind seemingly gets sucked in and I forget about everything else. I don’t remember, like even I lose the ego state, I guess… and I’m able to be completely absorbed in what I’m doing or in the story that’s going on. I wish I could get that state more often, bring that state out more often when I want to, rather than when I’m forced to, but… I like that hyperfocused state, if it’s applied to the correct thing.”

Participant 13: “So, yes. It’s maybe got your pros to… certain degrees. Some people will find something that they’re good at and… can focus on that and follow through with things. But they’re quite likely to then become hyperfocused and maybe miss other things that are going on around them. You know there’s the element of hyperactivity at times as well, just that too much excitement, when you get those little light bulb moments, which are great, but then actually remembering what they are later down the line, it’s not quite so easy”.

**Figure 1:**
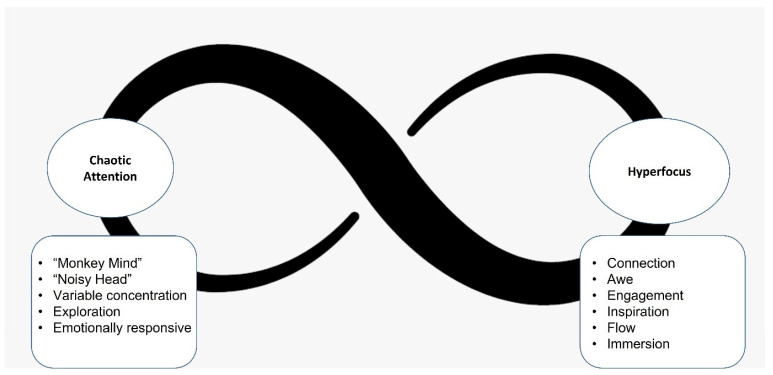
The polar experience of the states of ADHD consciousness.

Both *chaotic attention* and *hyperfocus* have been extensively described in the ADHD literature. What emerged from the data was how participants described themselves as experiencing a sense of movement both within and between these states. It was difficult for them to describe one state without relation to the other. Therefore, the model uses an infinity paradigm to capture the experience of ADHD consciousness (Figure 1).

### 3.3. Self-Regulation of ADHD Consciousness

**Figure 2:**
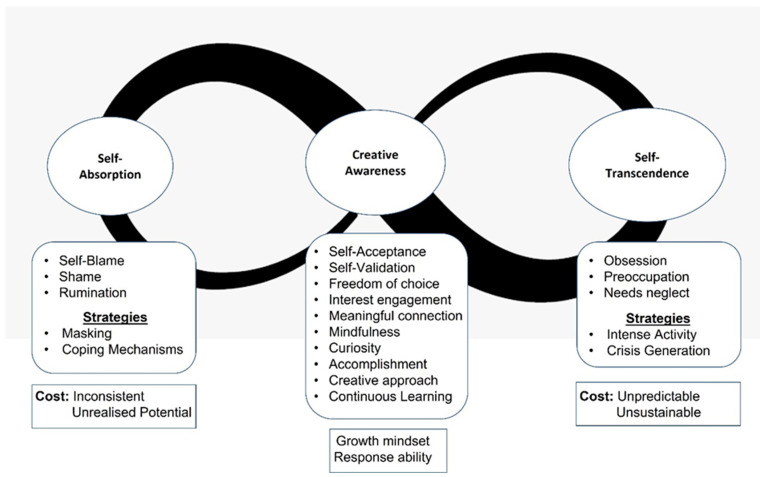
Polar model of ADHD self-regulation strategies for management of ADHD states of consciousness.

Self-regulation strategies demonstrate attempts at internalisation and active management of the states of ADHD consciousness. Three strategies for ADHD self-regulation emerged from the data: *self-absorption*, *self-transcendence*, and *creative awareness* (Figure 2).

### 3.4. Self-Absorption

*Self-absorption* strategies are primarily aimed at regulating *chaotic attention*. They are defined as “identifying the internal sense of self as both the origin and primary factor in self-regulatory control”. Participants described themselves as “dysfunctional”, “rubbish”, “broken”, “defective”, “deficient”, “pathetic”, “a failure” and having a sense of there being “something inherently wrong with me”. *Self-absorption* strategies are characterised by *self-blame, shame, and rumination*. Attempts to regulate *chaotic attention* centre on the assumption that the ADHD individual is at fault due to inherent flaws. *Self-absorption* strategies are accompanied by deep feelings of not belonging, worthlessness, unachieved potential, anxieties around rejection and lack of hope, faith in the future, and capability to achieve success. *Self-absorption* strategies are identified as *masking* and *coping mechanisms*. *Masking* is defined as “hiding behaviours or responses and presenting socially acceptable behaviours to facilitate belonging”. Participants described needing to “hide”, “pretend”, “pass as neurotypical” or “cover up” behaviours.

Participant 12: “So that’s the bit I have communicated. I haven’t told work. And there is a reason for that. I could do, but I haven’t. And the reason I haven’t is because quite a few years ago [while] I was still going through… the diagnosis… process, a new man came to work in our team and he was autistic. And there were lots of negative things said, and by senior management, you know,… not outright name calling, but I could see… that they treated them differently. It made me really—I don’t want them to do that to me. I can’t let them. It sounds like, you know that I’ve got this weakness, if that makes sense. I didn’t want to. I can deal with it myself… I can let a couple of close colleagues who I trust know, so that they can support me if I need it. So I made that decision”.

*Coping mechanisms* are defined as “strategies or behaviours used to reduce unpleasant emotions”. Also defined as “need substitutes” in SDT [73,78], examples of coping mechanisms emerging from the data are alcohol misuse, sugary food misuse, technology misuse, dependence on external individuals for organisation or accountability, and dependence on controlling external structures in the environment. Participants aimed to gain a sense of control by actively engaging in negative self-criticism, withdrawal and isolation, focusing on weaknesses, struggling to set boundaries, and attempts to “fix oneself”.

Participant 3: “I didn’t actually know what was going on in terms of my brain before I had that diagnosis. I actually was screened for a lot of different things before they came to the conclusion of ADHD because I was so clueless of what was going on. I think that was definitely a lot more struggle and unhealthy coping mechanisms going on. I had a lot of obsessive-compulsive tendencies, particularly before my diagnosis… and, I think I was… desperately trying to cope in the subtlest and quietest way possible, even at my own expense. Without these things in place, I would have felt a lot more lost and confused about what was going on. I just felt that there was something inherently wrong with me.”

While *self-absorption* strategies may assist with management of *chaotic attention* to some degree, they extract an energetic cost and impact on identity. They were described as inconsistent and exhausting, and participants did not feel they were being themselves or performing to their best.

### 3.5. Self-Transcendence

*Self-transcendence* strategies are primarily aimed at self-regulating *hyperfocus*. They are defined as “surrender of self-regulatory control to experience”. Participants described themselves as “drawn in”, “consumed”, “laser focused”, and the experience as, “nothing else mattered”, “couldn’t do anything else”, “too engaging”, “inhibiting” and “debilitating”. *Self-transcendence* strategies are characterised by *obsession*, *preoccupation* and *needs neglect*. Attempts to regulate *hyperfocus* centre on prioritising engagement as long as possible or until a task is complete or a problem solved, sometimes to the detriment of physical needs such as eating, sleeping or toileting. *Self-transcendence* strategies are accompanied by feelings of escapism, futility, pointless activity, narrow focus, engrossment, being stuck, and an inability to stop. *Self-transcendence* strategies are identified as *intense activity* and *crisis generation*. *Intense activity* is defined as “energetic involvement with experience”. Participants described feeling energised for the duration followed by exhaustion, succeeding in making progress and craving more of the experience.

Participant 7: “In one case… it was a small start-up, which we’ve grown, which meant that I needed to be able to for a couple of weeks do the impossible and basically not sleep and just hammer work. But I just was that engaged [it was just] a breeze. I was working probably, you know in some cases, two nights on the trot or two days on the trot without sleep through the night”.

*Crisis generation* is defined as “external pressure or internal feelings of anxiety, excitement, or stress which motivate engagement”. Participants expressed a need for external pressure, environmental crisis, or proximal deadlines to reduce options and utilise these strategies.

Participant 6: “So high pressure, for sure. Deadlines. In terms of uni work, the most practical thing I could give you now, my most intense work, most productive time, is when it comes to the point where if I don’t do it I won’t get done before the deadline; so when there are 16 h until the essay is due. Now we’re doing it. Or in emergencies or like crisis situations. If someone has, like, a medical emergency or something, I am my best self. I don’t know why I didn’t go into, like, paramedic—to be a paramedic as a career. I am just so focused on, like geared towards the situation. I feel like my best self… like I always feel really accomplished”.

While *self-transcendence* strategies assist with management of *hyperfocus*, they are chaotic in nature, extracting costs in energy and needs fulfilment. They were described as unpredictable and unsustainable, often occurring without conscious choice or control.

### 3.6. Creative Awareness

*Creative awareness* strategies appear at the centre of the infinity paradigm. They are defined as “open and receptive attention to the self and the environment”. Participants described themselves as “calm”, “passionate”, “naturally motivated”, “creative”, “playful”, “generating ideas”, and “engaged”. Creative awareness strategies are characterised by *self-acceptance*, or “an individual’s acceptance of positive and negative attributes”; *self-validation*, or “an individual’s acceptance of internal experience, including thoughts and feelings”; *freedom of choice*, or “an individual’s opportunity and autonomy to perform an action selected from at least two available options, unconstrained by external parties”; *interest engagement*, or “stimulated fully focused attention”; *meaningful connection*, or “in congruent relationship with an individual’s values”; *mindfulness*, or “wide flexible present moment awareness”; *curiosity*, or “interest leading to inquiry”; *accomplishment*, or “sense of achievement and capability”; *creative approach*, or “ability to respond to context with originality”; and *continuous learning*, or “desire to improve knowledge”. Participants described feeling a sense of ease and capability, where they can adapt to the context, identify options, and respond effectively and successfully. They experience a sense of enjoyment, resourcefulness, and an ability to apply skills and knowledge based on an acceptance and understanding of their internal experience. This includes an awareness of their own needs, and generates confidence to set boundaries, prioritise and engage with effective tools, and request support. Activities that require effort but are also meaningful generate positive feelings of satisfaction with a job well done. Opportunities to problem solve, generate solutions or “think outside the box” provide a chance to contribute in ways that have value.

Participant 1: “I would feel really bright, really optimistic, really sunny and really, full of excitement to deliver whatever I’m doing. Or just I suppose, in the flow of things, which is really exciting and feeling a part of something as well, rather than like a spare part. I will feel, not important in a grandiose way, but I will know that what I’m doing will be valued and is helpful and nobody else could do. That’s a really great feeling because it’s not like, oh, I’m doing this for praise or something like that. It’s just like, I am in the right hole. I’m not a square peg in a round hole, I’m in the right place. I’m in some place where I thrive”.

Participant 10: “I didn’t have a diagnosis actually until my adult years, but I always knew something was different. I didn’t realize I was doing it, but I was making coping mechanisms and things like that, you know, as I was going along, whilst being very true to myself anyway. I never got myself a deep dark rut or whatever, trying to hide who I was. I’d just try and put myself in a position where I was capable. So, no, I think I kind of get it. It’s the similar thought process I went through of, “it is what it is”. I just think about things differently”.

Participant 6: “Let it wash over. You try not to internalize it. Cause… we’ve grown up being frustrated with who we are. Like, why can’t you just be better? Right. Try not to make it your fault. And then think about accommodation. Think about what is wrong. What are the most impeding symptoms that you have in your life? Things that you don’t like to think about now, how you can change them because you tried neurotypical ways of solving problems in the past. You have obviously, you know… I said to the counsellors, like, I’ve tried everything, and nothing makes me feel better because I’m using the wrong solutions. I’m addressing the wrong problems”.

*Creative awareness* strategies are identified as having a *growth mindset*, or “believing inherent abilities and learned skills can be developed over time”, and *response ability*, or “the ability to respond to the present context”. Participants described both a desire and active effort to develop their self-knowledge and improve their skills and ability to be successful. They demonstrated strong resilience in the face of lifelong challenges, and a positive outlook for the potential future. Creative awareness strategies exemplify internalised identity commitments and perception of the AIC. Participants shared stories where their skills and abilities resolved situations or provided additional resources to facilitate positive outcomes. They described feeling present, focused, engaged, and able to meet challenges creatively and effectively.

## 4. Discussion

Participants were clear that experience of consciousness centred around the differences in engagement between *chaotic attention* and *hyperfocus* and the environment generate core perceptions of self. This changing sense of self in reference to the relationship between awareness and experience is echoed in the Buddhist perspective of “groundlessness” of consciousness [79], and is exemplified in ontological phenomenalism [80,81], the emergence of self in Gestalt therapy [82], the “middle mode” of embodied cognition [83] and the “self-as-process” of SDT [73,76].

### 4.1. Challenges with ADHD Self-Regulation and Identity Construction

**Figure 3:**
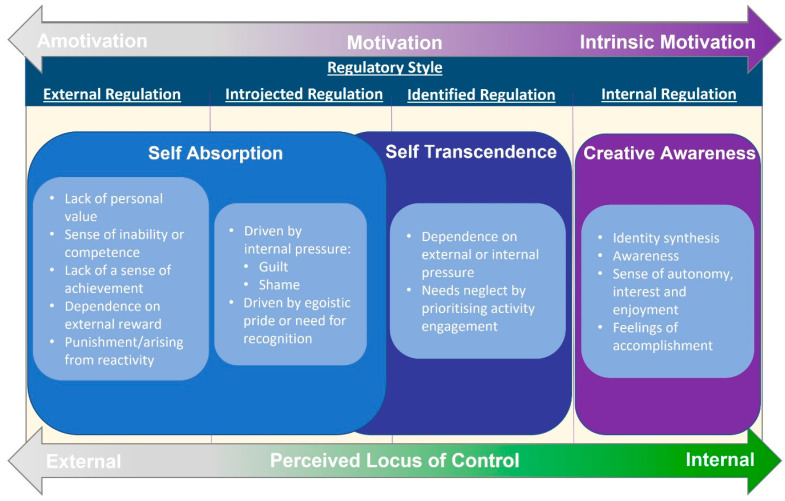
Creative Awareness Theory (CAT) and self-determination theory (SDT) regulatory styles.

*Self-absorption* and *self-transcendence* emerged from the data as unskilled strategic attempts at self-regulation and internalisation of the polar nature of ADHD consciousness. While these strategies may appear successful on the surface, the unreliability, lack of authenticity, and energetic cost demonstrated a clear negative long-term impact on participants’ self-concept and identity construction. From an SDT perspective, each strategy for behavioural regulation and motivation would be identified as originating from a different perceived locus of causality (PLOC) [84]. *Self-absorption* strategies characterised by lack of personal value, sense of inability or competence, and lack of a sense of achievement demonstrate a state of amotivation. Those characterised by dependence on external reward or punishment or arising from reactivity are externally regulated. Finally, strategies characterised by internal pressure such as guilt or shame and need for concealment or driven by egoistic pride or need for recognition represent introjected regulation. *Self-transcendence* strategies demonstrate more characteristics of interest, personal value, and congruence with outcomes; however, due to the nature of these strategies’ dependence on external or internal pressure and prioritisation of engagement with activity to the degree of neglecting needs, these strategies are identified as external or identified regulation. Because these strategies are more controlled, external, and low in autonomy (and therefore originate from goals adopted from outside the self [73,84]), they will be demonstrably less effective in ADHD self-regulation, and identity commitments will be less internalized (Figure 3).

### 4.2. Resources for ADHD Self-Regulation

Creative awareness strategies emerged from the data as participants’ description of awareness of the polar opposites of consciousness and self-regulation of that awareness. These skills in self-regulation exemplify Perls et al.’s [82] process of creative adjustment, based on Friedlander’s [85] philosophical concept of the polarity principle. Friedlander theorises that the basic characteristic of any phenomenon is that it can go to extremes. For that to be appreciated, it must be different from something else—namely its polar opposite [85,86,87]. Thus, the two polar extremes are internally connected. This polar opposition can only be distinguished through differentiation, or by considering one phenomenon relative to its polar opposite, e.g., it is relatively light compared to the polar opposite of dark [87]. The centre between these poles is the focus of Friedlander’s philosophy, what he calls its “indifference”:

“From time immemorial, when dealing with polarities. more attention has been paid to the poles than to the indifference. Yet in this indifference lies the real secret, the creative will, the polarizing one itself, which objectively is absolutely nothing. However, without indifference there would be no world” [87], (p.118).

This indifference is the creative centre of reality, from which differentiation into opposites takes place. This is not a static midpoint, however, but a lively multiplicity of differentiation of many polarities exemplified in Friedlander’s resistance to using a singular label or term and instead using variations such as ego, self, being, subject, individual, identity, person, mind, soul, absoluteness, ∞, insistence, will and freedom [87]. The aim of Friedlander’s philosophy is to achieve authentic creativity through indifferentiation of one’s own awareness, achieving an indifferent clarity of mind leading to integration and becoming centred. This is achieved through an active art of balance, or equilibration, where a person remains centred in indifference with an awareness of the link between opposites, allowing the freedom to react appropriately to the demands of a situation from either pole [87]. Perls [88] describes this equilibration as “differential thinking”, which forms the foundation for successful *creative adjustment* at the point of contact with the environment [82]. Personified as a creative and dynamic acceptance and assimilation of novelty resulting in growth, *creative adjustment* is seen as synonymous with *thriving*, or access to true self-regulation, in SDT [73]. From an SDT perspective, *creative awareness* strategies are characterised by a sense of self-direction, awareness, persistence, a sense of meaning, and vitality while engaging with challenges are all features of a perception of the AIC [89,90]. Combined with a sense of autonomy, interest and enjoyment, identity internalisation and feelings of accomplishment, these characteristics are all features of integrated and intrinsic regulation.

A review of the data also shows that the themes identified in *creative awareness* strategies also support the three basic psychological needs that must be satisfied to sustain psychological interest, wellness and development, defined in SDT as *autonomy*, *competence* and *relatedness* (Table 2) [73,91]. Therefore, participants’ experience of *creative awareness* strategies are identified in this analysis as the ADHD experience of SDT’s intrinsic regulation, internalisation, or effective self-regulation.

## 5. Limitations and Further Research

Charmaz’s [56] constructivist grounded theory methodology was specifically chosen for this project in light of the researcher’s lived experience of ADHD, which does present a risk of bias. The approach recognises the interpretive and reflexive role of the researcher, fostering awareness of presuppositions and interpretations and their effect on the research. As the theory is “dependent on the researcher’s view; it does not and cannot stand outside it” [56] (p. 239), the intention of this study was to view the experience of ADHD through the lens of ADHD with the aim of revealing new perspectives. Recent developments in neurodiversity activism have led to models of ‘inclusive research’ [92] which promote the contribution of neurodivergent researchers to the field [93,94,95]. A primary goal of this study was to contribute this viewpoint to the broader literature while simultaneously recognising the influence of this perspective.

In line with grounded theory methodology [56], saturation of data was identified at the completion of 11 interviews. As no new properties were identified, data were considered dense enough for theoretical completeness. While this may not be generalisable by the standards of qualitative research, grounded theory methodology supports quality of data from significant analysis despite a small sample size [96]. The findings from this study are important in exploring a different perspective on the experience of ADHD self-regulation that generates opportunities for increased self-understanding by individuals with ADHD.

We suggest that the CAT can be used in diverse settings to support individuals with ADHD in two areas: through psychoeducation, to better understand their experiences of engagement, and as a framework through which to develop the strategies demonstrated in *creative awareness* for self-regulation. The goals for professionals working with adults with ADHD should be to assist them to identify the impact that the experience of polarity has on self-belief, identity construction and self-concept; increase critical awareness of polarity strategies they are engaging with in a given context; and encourage the use of resources such as curiosity and mindfulness to increase flexibility and creativity in response. Further research is recommended to develop interventions to investigate the feasibility of the model as a basis for treatment. A novel 11-session therapeutic self-development, psychoeducation and skills training programme grounded in SDT and using the CAT has been piloted to examine participant acceptability, feasibility and efficacy. This programme uses a multi-modal psychotherapeutic approach and educational methodology to assist participants in understanding ADHD and developing self-regulation skills.

## 6. Conclusions

The ADHD Creative Awareness Theory (CAT) highlights the significance of the polar nature of the interaction of ADHD consciousness and the environment, identified in this study as *chaotic attention* and *hyperfocus*. The challenges of active management of this polar engagement alongside a lack of understanding lead to unskilled maladaptive self-regulation strategies, known as *self-absorption* and *self-transcendence*, which may allow an ADHD individual to operate at a functional level but come at a high cost to their energetic resources, self-concept, identity commitments, and fulfilment of needs. Recognition and understanding of the ADHD interactive polarity generates opportunities for effective self-regulation strategies, or *creative awareness*, which arise both naturally and through skill development from lived experience of ADHD. These strategies reflect the SDT perception of an authentic inner compass and definition of integrated or intrinsic self-regulation, and they contribute to a much-needed growing body of research on the positive human qualities of ADHD.

Recent research indicates that the understanding of ADHD self-regulation is dominated by cognitive behavioural theoretical perspectives [1]. These theories centralise executive function differences as impairing deficits, informing the definition of ADHD and specifying symptom reduction and behavioural control as primary treatment outcomes [54]. We propose that the CAT presents an emergent model of ADHD identity construction and self-regulation that provides both a positive perspective on the capability of individuals with ADHD to coherently self-regulate and a foundational skill set to generate more universal treatment outcomes.

## Figures and Tables

**Table 1:** Participant characteristics.

Patient ID	Age	Gender	Race	Year of Diagnosis	Subgroup
1	38	Female	White British	2020	Combined
3	21	Female	White British	2018	Combined
4	35	Male	White British	2011	Inattentive
6	20	Female	White British	2021	Combined
7	24	Male	White British	2009	Inattentive
8	20	Male	Hispanic American	2021	Inattentive
9	52	Female	White British	2019	Inattentive
10	28	Male	White British	2021	Inattentive
11	50	Male	South American	2016	Inattentive
12	42	Female	White British	2016	Inattentive
13	36	Female	White British	2013	Combined

**Table 2:** Core themes of creative awareness and SDT basic psychological needs.

SDT Basic Psychological Needs		
Autonomy	Competence	Relatedness
Self-acceptance Self-validation Freedom to choose Creative approach Interest engagement Curiosity	Accomplishment Continuous learning	Meaningful connection Mindfulness

## Data Availability

The data that support the findings will be available in PsychArchives at https://doi.org/10.23668/psycharchives.15430, following an embargo from the date of publication to allow for the commercialization of research findings.

## Supplementary Materials

The following supporting information can be downloaded at: https://www.mdpi.com/article/10.3390/jcm13195963/s1, Figure S1: Concept Map 1—Initial Concepts. Figure S2: Concept Map 2—Theoretical Concepts. Figure S3: Concept Map 3—Self-Focus Process Map. Figure S4: Concept Map 4—Self-Transcendence Process Map.
