# Supplementary material for: The Creative Awareness Theory: A Grounded Theory Study of Inherent Self-Regulation in Attention Deficit Hyperactivity Disorder
Source: J Clin Med. 2024 Oct 7;13(19):5963. doi: 10.3390/jcm13195963 (PMC11477866; doi:10.3390/jcm13195963)

Supplementary Material: Concept Maps for process development

Figure S1 - Concept Map 1: Initial concepts

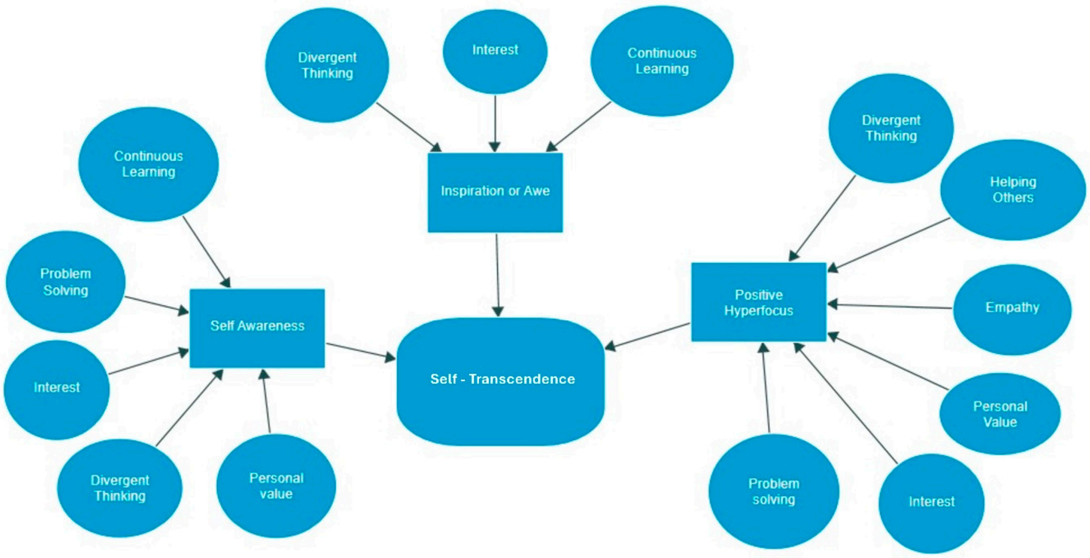

Figure S2 - Concept Map 2: Theoretical concepts

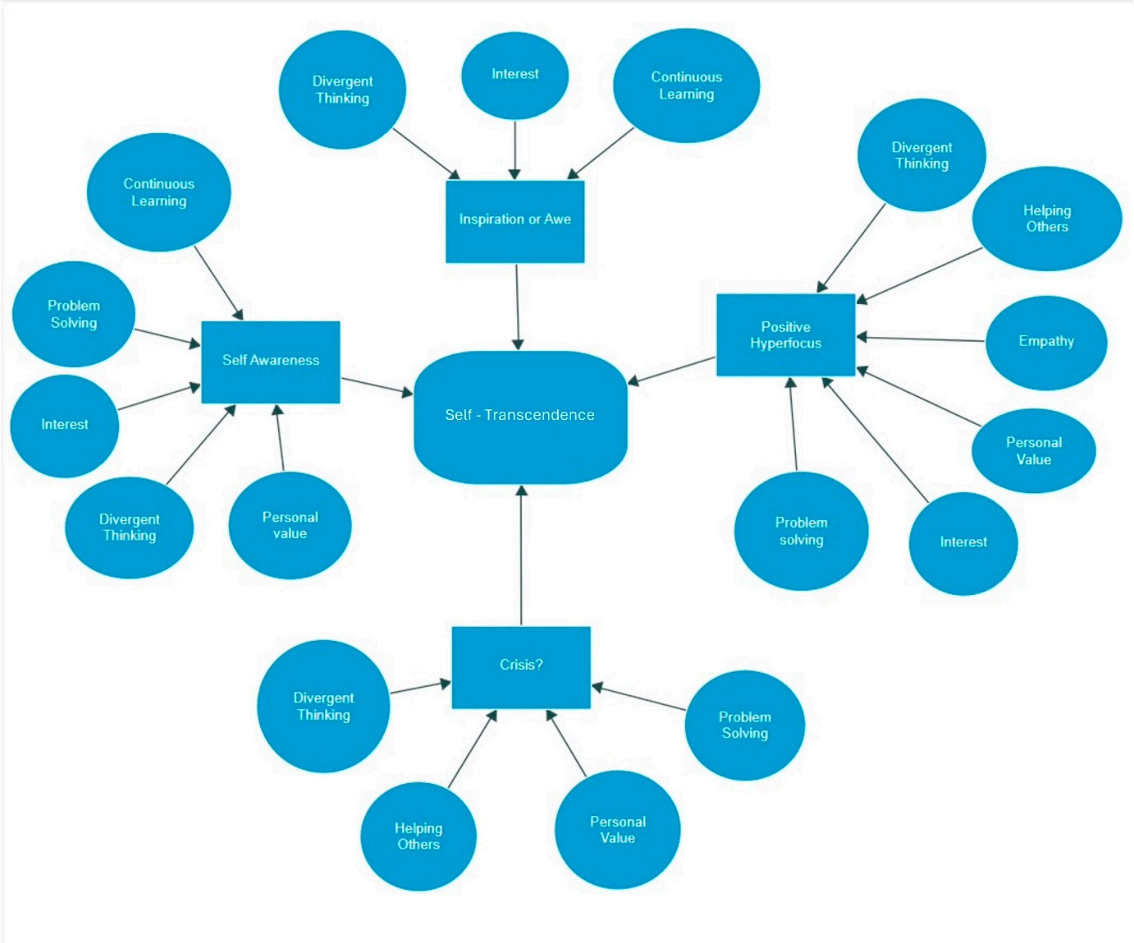

Figure S3- Concept Map 3: Self-Focus Process Map

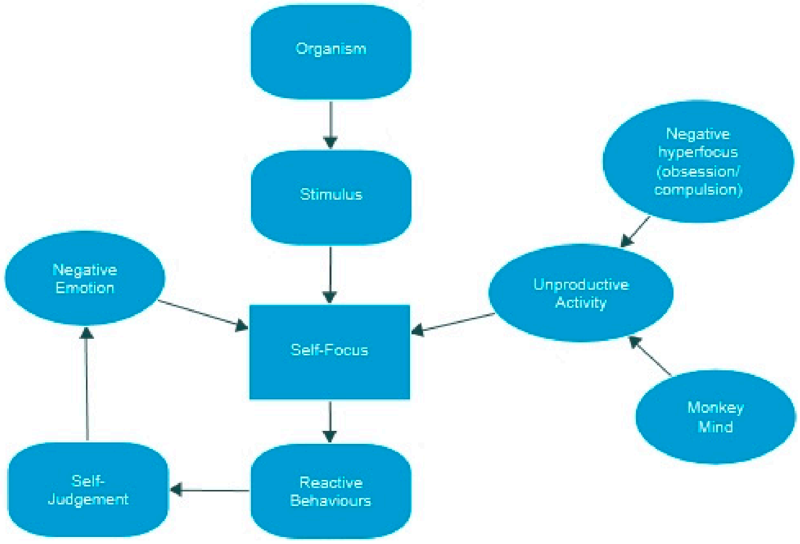

Figure S4 - Concept Map 4: Self-Transcendence Process Map

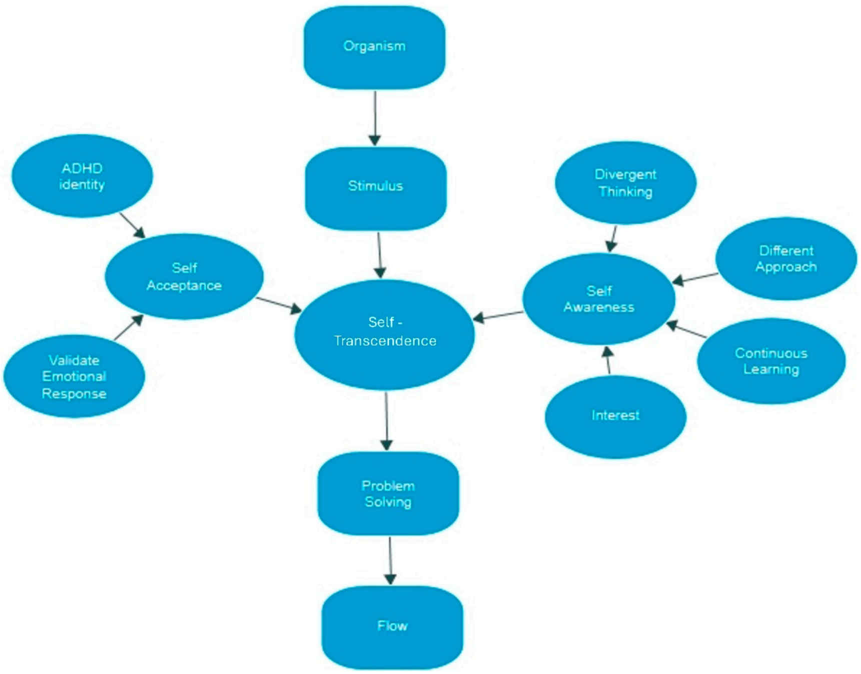

Supplement: Supplementary file 1 [file jcm-13-05963-s001.zip › jcm-3229222-supplementary.pdf]
